# Early urinary sodium vs B-type natriuretic peptide for predicting 30-day outcomes in acute decompensated heart failure: a prospective cohort study

**DOI:** 10.3325/cmj.2026.67.273

**Published:** 2026-08

**Authors:** Ahmet Cagdas Acara, Kursat Kaan Kerimoglu

**Affiliations:** 1 Emergency Department, Bozyaka State Hospital, Izmir, Türkiye; 2 Department of Emergency Medicine, Alsancak Nevvar-Salih Isgoren State Hospital, Izmir, Türkiye

## Abstract

**Aim:**

To compare the prognostic performance of early urinary sodium (UNa) and B-type natriuretic peptide (BNP) for predicting 30-day adverse outcomes in emergency department (ED) patients with acute decompensated heart failure (ADHF).

**Methods:**

This prospective observational study was conducted in the ED of Dokuz Eylül University Hospital, Izmir, between January 2025 and July 2025. The study enrolled adults presenting with dyspnea, diagnosed with ADHF, and treated with intravenous loop diuretics. Baseline and 2-hour spot UNa and serum BNP levels were measured. The primary outcome was a composite of intensive care unit admission at the index visit, ED revisit due to ADHF within 30 days, or 30-day all-cause mortality. Patients were compared according to the presence or absence of the composite outcome. Logistic regression and receiver operating characteristic analyses were performed.

**Results:**

A total of 117 patients were enrolled, and the composite outcome occurred in 41 (35.0%). Baseline BNP and UNa levels did not differ significantly between the groups. However, 2-hour UNa was significantly lower in patients with the composite outcome than in patients without it (88.0 ± 29.1 vs 107.3 ± 16.6 mmol/L, *P* < 0.001), whereas 2-hour BNP was comparable between the groups. In multivariable analysis, 2-hour UNa remained independently associated with the composite outcome (adjusted odds ratio 0.963; 95% confidence interval 0.942-0.986; *P* = 0.001), while 2-hour BNP did not. In receiver operating characteristic curve analysis, 2-hour UNa showed significant discriminative ability (area under the curve [AUC] 0.739), whereas 2-hour BNP did not (AUC 0.542). The AUC of 2-hour UNa was significantly greater than that of 2-hour BNP (*P* = 0.003).

**Conclusion:**

Lower 2-hour UNa was independently associated with 30-day adverse outcomes in ED patients with ADHF and may provide early prognostic information on natriuretic response. These findings require validation in larger multicenter studies.

Acute decompensated heart failure (ADHF) ranks among the primary causes of emergency department (ED) visits and hospital admissions globally, considerably contributing to short-term morbidity, mortality, and health care burden (1,2). Despite advancements in guideline-directed medical therapy, early post-discharge outcomes continue to be suboptimal, with a significant proportion of patients dying or requiring rehospitalization within 30 days (3). Consequently, there is a need to identify patients at risk of early clinical deterioration at ED presentation (4).

Intravenous loop diuretics are the fundamental component of initial therapy in ADHF (5). Because successful decongestion is strongly associated with improved clinical outcomes (6,7), an early assessment of diuretic effectiveness is clinically desirable (7). However, during the first hours after therapy initiation, conventional indicators of treatment response, including symptomatic relief, body weight, or physical examination findings, are often delayed or insufficiently sensitive (7,8). Consequently, it is important to identify objective early biomarkers that reflect diuretic responsiveness and predict short-term prognosis (9).

B-type natriuretic peptide (BNP) is an established biomarker used in the diagnosis and risk stratification of heart failure (10). Baseline BNP concentrations correlate with disease severity and long-term prognosis (11), while serial measurements during hospitalization may provide additional prognostic information (12). However, BNP predominantly reflects myocardial wall stress and neurohormonal activation rather than the immediate effectiveness of decongestive therapy (13). Accordingly, its ability to capture very early treatment response during the first hours after intravenous diuretic administration remains unclear.

In contrast, urinary sodium (UNa) has emerged as a promising functional marker of early diuretic response (14). By directly reflecting natriuresis induced by loop diuretics, UNa provides an immediate assessment of renal responsiveness to decongestive therapy (15). Previous studies have shown reduced post-diuretic UNa to be associated with impaired diuretic efficiency, persistent congestion, prolonged hospitalization, and worse in-hospital outcomes (16-18). While BNP reflects cardiac wall stress, UNa reflects the pharmacodynamic effect of treatment at the kidney level, which suggests that these biomarkers provide complementary rather than competing physiological information (19).

Although the prognostic significance of post-diuretic UNa has been increasingly recognized, most previous studies have focused on hospitalized patients and in-hospital outcomes (16-18). It remains unknown whether an early ED-based UNa assessment provides better short-term prognostic information than contemporaneously measured BNP. Therefore, this prospective observational study compared the prognostic performance of baseline and 2-hour UNa and BNP measurements for predicting 30-day adverse outcomes in ED patients with ADHF receiving intravenous loop diuretics. We hypothesized that the early natriuretic response, reflected by 2-hour UNa, would provide better prognostic information for short-term clinical deterioration than early changes in BNP.

## Patients and methods

### Study design

This prospective observational study was conducted in the ED of Dokuz Eylül University Hospital, a tertiary care hospital in Izmir, Turkey, between January 2025 and July 2025. Consecutive adult patients presenting with dyspnea and diagnosed with ADHF, classified as New York Heart Association (NYHA) functional class III or IV, and treated with intravenous loop diuretics for decongestion, were screened for eligibility.

### Study outcomes

The primary outcome was a composite endpoint consisting of admission to the intensive care unit (ICU) during the initial visit, ED revisit due to ADHF within 30 days, or all-cause mortality within 30 days. These outcomes were chosen due to their representation of clinically significant manifestations of early disease progression or failure of initial management following ED presentation. ICU admission reflects severe clinical deterioration requiring escalation of care during the index visit, whereas 30-day ED revisit and mortality reflect unsuccessful early stabilization after discharge.

### Sample size and study participants

Patients were eligible for inclusion if they presented to the ED with dyspnea, had a preliminary diagnosis of acute heart failure, were classified as NYHA class III-IV, and were initiated on intravenous loop diuretic therapy. Patients were excluded if they had another diagnosis that could explain dyspnea; were on routine hemodialysis; had ADHF secondary to acute coronary syndrome; were diagnosed with cardiogenic shock; could not be clinically followed during the first 2 hours because of death or transfer; or had received intravenous loop diuretic treatment before collection of the baseline urine sample. Patients who declined participation were also excluded. Patients with multiple ED visits were included only once. In total, 117 patients were enrolled.

Sample size was calculated using OpenEpi (https://www.openepi.com/Menu/OE_Menu.htm) based on the expected composite adverse outcome rate reported by Xanthopoulos et al (20) in a prospective cohort of patients with ADHF. Assuming an expected event rate of 50%, a 95% confidence level, and a margin of error of 10%, the minimum required sample size was 97 patients.

### Data collection

We recorded demographic characteristics, comorbidities, vital signs at admission and at 2 hours, Glasgow Coma Scale score, NYHA functional class, dyspnea severity, clinical congestion-perfusion profile, laboratory findings, echocardiographic parameters, treatments administered in the ED, and clinical outcomes. Data were obtained from the hospital information management system and entered into a standardized study form.

Dyspnea severity was assessed using a 100-mm visual analog scale (VAS). Patients were asked to indicate the severity of their current breathing difficulty by marking a point on a horizontal or vertical line, and the score was calculated by measuring the distance in millimeters from the starting point to the marked point.

All enrolled patients underwent Foley catheterization. Immediately after catheter placement, the initial bladder urine drained was recorded as the baseline urine output. A portion of this urine was collected for baseline UNa and urinary chloride analysis before administration of intravenous furosemide. Patients in whom intravenous loop diuretic therapy had been initiated before the collection of the baseline urine sample were not included in the study. Following baseline urine sampling, intravenous furosemide treatment was started. Urine output during the first 2 hours after furosemide administration was measured as the total urine volume accumulated in the urine bag during this period. At the end of the second hour, the Foley catheter tubing was briefly clamped to measure urine output and collect a spot urine sample. The second-hour urine sample was obtained from the newly accumulated urine in the catheter tubing. UNa and chloride were measured at baseline and at 2 hours.

The baseline evaluation included clinical assessment, blood and urine laboratory testing, and echocardiographic examination. BNP levels were measured using the ARCHITECT ci16200 Integrated System (Abbott Diagnostics, Abbott Park, IL, USA). UNa and urinary chloride levels were assessed using the AU5800 clinical chemistry analyzer (Beckman Coulter, Brea, CA, USA). Echocardiographic examinations were performed using a Venue point-of-care ultrasound system (GE HealthCare, Chicago, IL, USA). Echocardiographic data included ejection fraction and venous excess ultrasound (VExUS) classification (21). VExUS grading was used to assess systemic venous congestion based on inferior vena cava assessment together with Doppler evaluation of the hepatic, portal, and intrarenal veins. During ED follow-up, treatments including total furosemide dose, intravenous nitrate administration, noninvasive mechanical ventilation, and intravenous inotropic support were recorded.

Patients were followed for 30 days after discharge. In-hospital outcomes and 30-day survival status were assessed by the study team using the hospital information management system and the national electronic health record system (e-Nabiz). For patients whose outcome data could not be obtained from these sources, follow-up information was collected by telephone contact.

The study was approved by the Dokuz Eylül University Non-Interventional Research Ethics Committee. Written informed consent was obtained from patients or, when applicable, from their first-degree relatives before study enrollment.

### Statistical analysis

The distribution of continuous variables was assessed using histograms, Q-Q plots, and the Shapiro-Wilk test. Continuous variables are presented as means ± standard deviations or as medians (interquartile ranges). Categorical variables are presented as numbers and percentages. Differences in categorical variables were assessed with a χ^2^ test or a Fisher exact test. Differences in continuous variables were assessed with a *t* test or a Mann-Whitney U test. Univariable logistic regression analysis was performed to evaluate the association between independent variables and the composite outcome. Variables showing a significant association in univariable analysis and/or considered clinically relevant were entered into a multivariable logistic regression model. A parsimonious model was constructed considering the number of outcome events and potential collinearity between related variables. Two-hour BNP was retained in the model as a predefined comparator biomarker. Odds ratios and 95% confidence intervals were calculated.

Receiver operating characteristic (ROC) curve analysis was used to assess the discriminatory performance of selected biomarkers for predicting the composite outcome, and the area under the curve (AUC) with 95% confidence intervals was calculated. Optimal cutoff values were determined using the Youden index. The AUCs of 2-hour UNa and 2-hour BNP were compared using the DeLong test. For the selected cutoff values, diagnostic and predictive performance measures, including sensitivity, specificity, positive predictive value, negative predictive value, relative risk, odds ratio, positive likelihood ratio, negative likelihood ratio, and overall accuracy, were calculated with 95% confidence intervals. A two-sided p value of <0.05 was considered statistically significant. Statistical analysis was performed using SPSS Statistics for Windows, version 26.0 (IBM Corp., Armonk, NY, USA) and MedCalc Statistical Software (MedCalc Software Ltd, Ostend, Belgium).

## RESULTS

The study enrolled 117 patients with a median age of 75.0 years (IQR, 67.0-81.5); 53.8% (n = 63) were male. Fifty patients (42.7%) were discharged from the ED, 48 (41.0%) were admitted to the ward, 17 (14.5%) were admitted to the ICU, and 2 (1.7%) died in the ED (Table 1). During the 30-day follow-up, 23 patients (19.7%) revisited the ED because of ADHF and 13 patients (11.1%) died from any cause. The composite outcome, defined as ICU admission at the initial presentation, ED revisit due to ADHF within 30 days, or 30-day all-cause mortality, occurred in 41 patients (35.0%) (Table 1). Because multiple components of the composite endpoint occurred in some patients, the total number of composite outcome events was lower than the sum of their individual components. Patients with and without the composite outcome were comparable in terms of age, sex, baseline comorbidities, and electrocardiographic findings (Table 2).

**Table 1:** Emergency department (ED) disposition and 30-day outcomes of the study population*

Outcomes	No.	%
ED		
discharged from the ED	50	42.7
admitted to the ward	48	41.0
admitted to the ICU	17	14.5
died in the ED	2	1.7
30-d		
revisit due to ADHF	23	19.7
all-cause mortality	13	11.1
Composite^†^	41	35.0

*Abbreviations: ICU – intensive care unit; ADHF – acute decompensated heart failure.

†The composite outcome was defined as ICU admission, 30-day revisit due to ADHF, or 30-day all-cause mortality.

**Table 2:** Baseline characteristics in patients with and without the composite outcome

Characteristics^§^	Total (n = 117)	Composite outcome		*P* value
		no (n = 76)	yes (n = 41)	
Age, median (IQR)	75.0 (67.0-81.5)	75.0 (67.0-80.0)	75.0 (67.0-83.5)	0.886^‡^
Sex				
female	54 (46.2)	34 (44.7)	20 (48.8)	0.676*
male	63 (53.8)	42 (55.3)	21 (51.2)	
Comorbid diseases				
coronary artery disease	78 (66.7)	55 (72.4)	23 (56.1)	0.075*
hypertension	69 (59.0)	42 (55.3)	27 (65.9)	0.267*
diabetes	43 (36.8)	27 (35.5)	16 (39.0)	0.708*
atrial fibrillation	33 (28.2)	22 (28.9)	11 (26.8)	0.808*
ischemic heart disease	24 (20.5)	17 (22.4)	7 (17.1)	0.499*
dyslipidemia	12 (10.3)	7 (9.2)	5 (12.2)	0.612*
malignancy	6 (5.1)	3 (3.9)	3 (7.3)	0.430^†^
other	21 (17.9)	12 (15.8)	9 (22.0)	0.407*
Electrocardiography findings				
normal sinus rhythm	41 (35.0)	27 (35.5)	14 (34.1)	0.881*
atrial fibrillation	33 (28.2)	22 (28.9)	11 (26.8)	0.808*
sinus tachycardia	23 (19.7)	16 (21.1)	7 (17.1)	0.606*
bundle branch block	11 (9.4)	8 (10.5)	3 (7.3)	0.572^†^
other	9 (7.6)	3 (3.9)	6 (14.6)	0.039^†^

*χ^2^ test.

†Fisher exact test.

‡Mann-Whitney U test.

§Unless otherwise specified, values are presented as n (%).

Assessment of vital signs at admission and at 2 hours showed that only heart rate at presentation was significantly higher in patients who developed the composite outcome than in those who did not (103.2 ± 14.1 vs 97.5 ± 15.0 beats/min, *P* = 0.048). The groups did not significantly differ in systolic or diastolic blood pressure, respiratory rate, or oxygen saturation. Among the clinical hemodynamic profiles, the wet-cold phenotype was significantly more frequent in the composite outcome group (26.8% vs 10.5%, *P* = 0.023). The groups did not differ significantly in terms of NYHA functional class, dyspnea VAS score, and duration of heart failure (Table 3).

**Table 3:** Vital signs, clinical scores, and duration of heart failure in patients with and without the composite outcome*

Characteristics		Total (n = 117)	Composite outcome		*P* value
			no (n = 76)	yes (n = 41)	
Vital signs					
SBP, mean (SD)	admission	148.6 ± 20.2	146.4 ± 18.5	152.7 ± 22.7	0.110^§^
	2 h	134.6 ± 19.4	132.3 ± 18.9	138.9 ± 20.0	0.084^§^
DBP, mean (SD)	admission	85.9 ± 14.0	84.6 ± 13.2	88.3 ± 15.3	0.170^§^
	2 h	77.2 ± 13.9	75.8 ± 13.2	79.7 ± 14.8	0.150^§^
heart rate, mean (SD)	admission	99.5 ± 14.9	97.5 ± 15.0	103.2 ± 14.1	0.048^§^
	2 h	84.0 ± 11.4	82.9 ± 11.8	86.1 ± 10.6	0.143^§^
RR, mean (SD)	admission	23.7 ± 6.5	23.5 ± 5.9	24.0 ± 7.7	0.692^§^
	2 h	18.6 ± 6.2	18.2 ± 5.8	19.6 ± 7.0	0.248^§^
SpO_2_, median (IQR)	admission	91.0 (88.0-95.0)	91.0 (88.0-94.0)	92.0 (88.0-95.5)	0.682^‡^
	2 h	95.0 (93.0-97.0)	95.0 (93.0-97.0)	94.0 (92.0-97.0)	0.071^‡^
Clinical hemodynamic profile, n (%)					
wet-warm		53 (45.3)	35 (46.1)	18 (43.9)	0.824^†^
dry-warm		27 (23.1)	19 (25.0)	8 (19.5)	0.501^†^
wet-cold		19 (16.2)	8 (10.5)	11 (26.8)	0.023^†^
dry-cold		18 (15.4)	14 (18.4)	8 (9.8)	0.215^†^
NYHA functional class, n (%)					
III		86 (73.5)	60 (78.9)	26 (63.4)	0.070^†^
IV		31 (26.5)	16 (21.1)	15 (36.6)	
Dyspnea VAS score, median (IQR)		70.0 (60.0-80.0)	70.0 (60.0-80.0)	70.0 (50.0-85.0)	0.686^‡^
Heart failure duration in years, median (IQR)		5.0 (3.0-7.0)	5.0 (3.0-7.0)	4.0 (2.0-6.5)	0.179^‡^

*SBP – systolic blood pressure; DBP – diastolic blood pressure; RR – respiratory rate; SpO_2_ – oxygen saturation; NYHA – New York Heart Association; VAS – visual analog scale.

†χ^2^ test.

‡Mann-Whitney U test.

§t-test.

Laboratory analyses revealed no significant differences in BNP levels (858.0 pg/mL vs 713.5 pg/mL, *P* = 0.504; and 831.0 pg/mL vs 675.5 pg/mL, *P* = 0.451, respectively) or high-sensitivity cardiac troponin I (hs-cTnI) levels between the groups, either at baseline or at 2 hours. In contrast, baseline serum lactate was significantly higher among patients with the composite outcome (2.1 vs 1.7 mmol/L, *P* = 0.013) (Table 4).

**Table 4:** Laboratory and renal findings in patients with and without the composite outcome*

Characteristics		Total (n = 117)	Composite outcome		*P* value
			no (n = 76)	yes (n = 41)	
Blood analysis					
BNP in pg/mL, median (IQR)	baseline	791.0 (432.0-1271.0)	713.5 (425.2-1216.0)	858.0 (456.5-1391.5)	0.504^†^
	2 h	736.0 (455.0-1184.5)	675.5 (429.0-1180.5)	831.0 (464.0-1190.5)	0.451^†^
hs-cTnI in ng/L, median (IQR)	baseline	18.1 (11.5-29.6)	18.5 (11.3-27.1)	18.0 (12.3-39.8)	0.178^†^
	2 h	20.3 (13.5-33.6)	20.5 (11.5-32.5)	19.8 (15.0-41.4)	0.243^†^
serum sodium in mmol/L, mean (SD)		135.7 ± 4.6	136.0 ± 4.7	135.2 ± 4.4	0.353^‡^
serum chloride in mmol/L, mean (SD)		99.9 ± 4.7	100.1 ± 5.0	99.5 ± 4.0	0.482^‡^
serum lactate in mmol/L, median (IQR)		1.9 (1.3-2.4)	1.7 (1.1-2.3)	2.1 (1.5-2.6)	0.013^†^
Urinary analysis					
urine sodium in mmol/L, mean (SD)	baseline	50.9 ± 28.9	53.8 ± 26.6	45.4 ± 32.5	0.133^‡^
	2 h	100.5 ± 23.6	107.3 ± 16.6	88.0 ± 29.1	<0.001^‡^
urine chloride in mmol/L, median (IQR)	baseline	52.0 (35.0-71.0)	53.0 (37.2-75.0)	52.0 (32.0-66.5)	0.517^†^
	2 h	85.0 (71.0-103.0)	89.5 (73.7-104.0)	78.0 (64.5-97.5)	0.035^†^
Renal function					
creatinine in mg/dL, mean (SD)		1.47 ± 0.5	1.45 ± 0.5	1.51 ± 0.5	0.621^‡^
BUN in mg/dL, median (IQR)		34.0 (23.5-44.0)	33.5 (22.7-44.0)	34.0 (23.5-44.5)	0.938^†^
eGFR in mL/min/1.73 m^2^, mean (SD)		50.7 ± 20.2	51.9 ± 20.6	48.6 ± 19.5	0.411^‡^
urine output in mL, median (IQR)	baseline	180.0 (100.0-250.0)	180.0 (100.0-250.0)	180.0 (100.0-250.0)	0.670^†^
	2 h	1400.0 (1000.0-1700.0)	1350.0 (950.0-1650.0)	1600.0 (1125.0-1800.0)	0.116^†^

*Abbreviations: BNP – B-type natriuretic peptide; hs-cTnI – high-sensitivity cardiac troponin I; BUN – blood urea nitrogen; eGFR – estimated glomerular filtration rate.

†Mann-Whitney U test.

‡t-test.

Regarding urinary biomarkers, baseline UNa levels were similar in the two groups (45.4 ± 32.5 mmol/L vs 53.8 ± 26.6 mmol/L, *P* = 0.133). However, 2-hour UNa was significantly lower in patients with the composite outcome than in those without it (88.0 ± 29.1 mmol/L vs 107.3 ± 16.6 mmol/L, *P* < 0.001). Similarly, 2-hour urinary chloride levels were significantly lower in the composite outcome group (78.0 vs 89.5 mmol/L, *P* = 0.035). The groups did not significantly differ in creatinine, blood urea nitrogen, estimated glomerular filtration rate, or urine output at baseline and at 2 hours (Table 4).

Echocardiographic assessment showed no significant difference in ejection fraction between the groups (40% vs 35%, *P* = 0.517). In contrast, the VExUS grade 3 was significantly more frequent in patients with the composite outcome (19.5% vs 6 *P* = 0.021). Regarding treatments administered in the ED, total furosemide dose and intravenous nitrate use were comparable between the groups. Inotropic support was required more often in the composite outcome group, with the difference reaching borderline significance (5 vs 2 patients, *P* = 0.050). Noninvasive mechanical ventilation was also used more frequently in the composite outcome group, although this difference did not reach significance (6 vs 3 patients, *P* = 0.064) (Table 5).

**Table 5:** Emergency department (ED) treatment and echocardiographic findings between patients with and without the composite outcome*

Characteristics	Total (n = 117)	Composite outcome		*P* value
		no (n = 76)	yes (n = 41)	
ED treatment				
total furosemide dose in mg, median (IQR)	100 (80-160)	80 (80-150)	120 (80-160)	0.444^‡^
intravenous nitrate use, n (%)	35 (29.9)	19 (25.0)	16 (39.0)	0.114^§^
inotrope requirement, n (%)	7 (6.0)	2 (2.6)	5 (12.2)	0.050^†^
NIMV requirement, n (%)	9 (7.7)	3 (3.9)	6 (14.6)	0.064^†^
ECO findings				
ejection fraction, median (IQR)	35 (30-45)	35 (30-45)	40 (30-45)	0.517^‡^
VExUS classification, n (%)				
grade 0	11 (9.4)	10 (13.2)	1 (2.4)	0.021^§^
grade 1	51 (43.6)	37 (48.7)	14 (34.1)	
grade 2	42 (35.9)	24 (31.6)	18 (43.9)	
grade 3	13 (11.1)	5 (6.6)	8 (19.5)	

*Abbreviations: ECO – echocardiography; NIMV – non-invasive mechanical ventilation; VExUS – venous excess ultrasound.

†Fisher exact test.

‡Mann-Whitney U test.

§χ^2^ test.

Univariable logistic regression analysis identified lower 2-hour oxygen saturation, higher admission hs-cTnI and serum lactate levels, lower 2-hour UNa and chloride levels, and higher VExUS grade as significant correlates of the composite outcome (Table 6). Of these, 2-hour UNa showed the strongest association (OR 0.960; 95% CI 0.941-0.980; *P* < 0.001), whereas 2-hour BNP was not significantly associated with the outcome (Table 6). In multivariable logistic regression analysis, a parsimonious model was constructed by including clinically relevant variables associated with the composite outcome in univariable analysis and avoiding redundancy among closely related predictors. Two-hour BNP was retained in the model as a predefined comparator biomarker, although it was not significantly associated with the outcome in univariable analysis, whereas 2-hour urinary chloride was not included because it represented a closely related post-diuretic urinary electrolyte response to UNa. The overall model was significant (omnibus *P* < 0.001) and showed acceptable explanatory performance (Nagelkerke R^2^ = 0.466), with an overall classification accuracy of 81.2% (Table 7). In the adjusted model, the 2-hour UNa level remained independently associated with the composite outcome, whereas the 2-hour BNP did not show an independent association (Table 7).

**Table 6:** Univariable logistic regression model for predicting the composite outcome*

Predictor	B	Standard error	Odds ratio	95% confidence interval	*P*-value
**Age**	0.001	0.017	1.001	0.969-1.034	0.936
**Sex**	−0.163	0.388	0.850	0.387-1.820	0.676
**SBP at admission**	0.015	0.010	1.015	0.966-1.035	0.113
**DBP at admission**	0.019	0.014	1.019	0.992-1.047	0.171
**RR at admission**	0.012	0.029	1.012	0.955-1.072	0.689
**HR at admission**	0.026	0.013	1.026	1.000-1.053	0.052
**SpO_2_ at admission**	−0.024	0.024	0.976	0.932-1.022	0.301
**SBP at 2 h**	0.017	0.010	1.017	0.998-1.038	0.087
**DBP at 2 h**	0.020	0.014	1.020	0.993-1.049	0.151
**RR at 2 h**	0.035	0.030	1.036	0.976-1.099	0.248
**HR at 2 h**	0.025	0.017	1.026	0.991-1.061	0.144
**SpO_2_ at 2 h**	−0.141	0.059	0.868	0.773-0.975	0.017
**Dyspnea VAS score**	−0.004	0.009	0.996	0.978-1.015	0.696
**hs-cTnI at admission**	0.019	0.009	1.020	1.002-1.038	0.032
**hs-cTnI at 2 h**	0.015	0.009	1.015	0.997-1.033	0.097
**Serum sodium**	−0.039	0.042	0.962	0.886-1.044	0.351
**Serum chloride**	−0.029	0.041	0.971	0.897-1.053	0.479
**Serum lactate**	0.600	0.219	1.823	1.186-2.803	0.006
**Serum creatinine**	0.164	0.329	1.178	0.618-2.246	0.618
**Serum BUN**	−0.004	0.008	0.996	0.981-1.012	0.662
**eGFR**	−0.008	0.010	0.992	0.973-1.012	0.408
**Urine output at admission**	−0.001	0.002	0.999	0.995-1.002	0.479
**Urine output at 2 h**	0.000	0.000	1.000	0.999-1.001	0.633
**Total furosemide dose**	0.003	0.004	1.003	0.995-1.011	0.458
**Serum BNP at admission**	0.000	0.000	1.000	1.000-1.001	0.355
**Serum BNP at 2 h**	0.000	0.000	1.000	1.000-1.001	0.210
**Urine sodium at admission**	−0.011	0.007	0.989	0.976-1.003	0.135
**Urine sodium at 2 h**	−0.041	0.010	0.960	0.941-0.980	<0.001
**Urine chloride at admission**	−0.005	0.008	0.995	0.979-1.011	0.507
**Urine chloride at 2 h**	−0.019	0.009	0.982	0.965-0.999	0.036
**Ejection fraction**	0.016	0.023	1.016	0.971-1.063	0.500
**VExUS grade**	0.788	0.263	2.200	1.313-3.685	0.003

*B – regression coefficient; SBP – systolic blood pressure; DBP – diastolic blood pressure; RR – respiratory rate; HR – heart rate; SpO_2_ – oxygen saturation; VAS – visual analog scale; hs-cTnI – high-sensitivity cardiac troponin I; BUN – blood urea nitrogen; eGFR – estimated glomerular filtration rate; BNP – B-type natriuretic peptide; VExUS – venous excess ultrasound.

**Table 7:** Multivariable logistic regression model for predicting the composite outcome*

Predictor	B	Standard error	Adjusted odds ratio	95% confidence interval	*P*-value
**SpO_2_ at 2 h**	−0.192	0.079	0.825	0.707-0.963	0.015
**hs-cTnI at admission**	0.022	0.010	1.022	1.003-1.042	0.025
**Serum lactate**	0.910	0.306	2.484	1.364-4.523	0.003
**Serum BNP at 2 h**	0.000	0.000	1.000	0.999-1.001	0.810
**Urine sodium at 2 h**	−0.037	0.012	0.963	0.942-0.986	0.001
**VExUS grade**	1.006	0.331	2.734	1.428-5.232	0.002

*Abbreviations: B – regression coefficient; SpO_2_ – oxygen saturation; hs-cTnI – high-sensitivity cardiac troponin I; BNP – B-type natriuretic peptide; VExUS – venous excess ultrasound.

**Figure 1:**
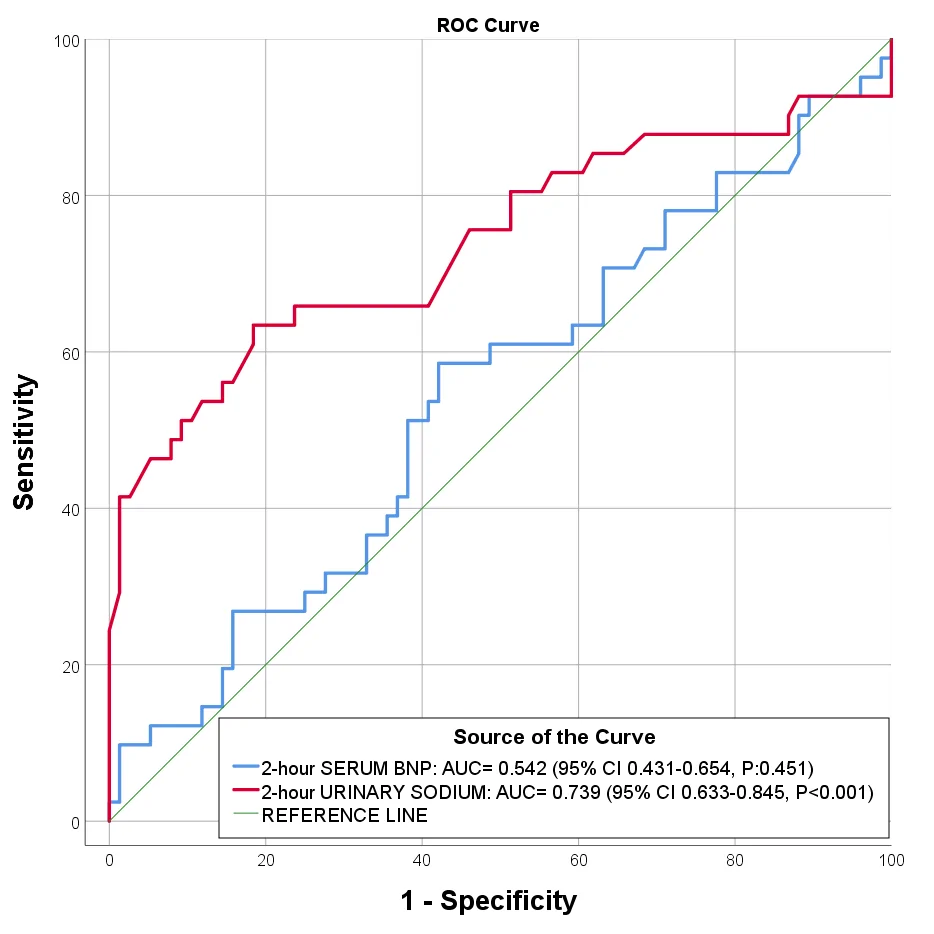
Receiver operating characteristic (ROC) curves of 2-hour urinary sodium and 2-hour B-type natriuretic peptide (BNP) for predicting the 30-day composite outcome.

In receiver operating characteristic curve analysis, 2-hour UNa demonstrated significant discriminative ability for the composite outcome, with an AUC of 0.739 (95% CI 0.633-0.845; *P* < 0.001), whereas 2-hour BNP did not show significant prognostic performance (AUC 0.542; 95% CI 0.431-0.654; *P* = 0.451) (Figure 1). The exploratory optimal cutoff value for 2-hour UNa was ≤93 mmol/L. The AUC of 2-hour UNa was significantly greater than that of 2-hour BNP (difference between AUCs 0.197; 95% CI 0.065-0.328; *P* = 0.003, DeLong test). At this cutoff, 2-hour UNa yielded a sensitivity of 63.4%, specificity of 81.5%, positive predictive value of 65.0%, and negative predictive value of 80.5%, with an overall accuracy of 75.2%. By comparison, 2-hour BNP had a sensitivity of 58.5%, specificity of 57.8%, and an accuracy of 58.1% (Table 8).

**Table 8:** Predictive performance of 2-hour serum B-type natriuretic peptide and 2-hour urinary sodium for 30-day composite outcome*

Parameter	2-h serum BNP	2-h urine sodium
Sensitivity (%, 95% CI)	58.5 (42.1-73.6)	63.4 (46.9-77.8)
Specificity (%, 95% CI)	57.8 (46.0-69.1)	81.5 (71.0-89.5)
Positive predictive value (%, 95% CI)	42.8 (34.1-52.0)	65.0 (52.2-75.8)
Negative predictive value (%,95% CI)	72.1 (63.1-79.6)	80.5 (73.1-86.2)
Relative risk (95% CI)	1.5 (0.9-2.5)	3.3 (2.0-5.5)
Odds ratio (95% CI)	1.9 (0.8-4.1)	7.6 (3.2-18.1)
Positive likelihood ratio (95% CI)	1.3 (0.9-2.0)	3.4 (2.0-5.8)
Negative likelihood ratio (95% CI)	0.7 (0.4-1.0)	0.4 (0.2-0.6)
Accuracy (%, 95% CI)	58.1 (48.6-67.1)	75.2 (66.3-82.7)

*Abbreviations: BNP – B-type natriuretic peptide; CI – confidence interval.

## DISCUSSION

In this prospective observational study of ED patients with ADHF treated with intravenous loop diuretics, 2-hour UNa level was independently associated with the 30-day composite outcome and showed higher discriminatory performance than 2-hour BNP. While baseline BNP and UNa values were not significantly different between the groups, patients who developed the composite outcome had markedly lower 2-hour UNa levels after initiation of intravenous loop diuretic therapy. In contrast, 2-hour BNP was not significantly associated with the outcome in either ROC or adjusted regression analyses. These findings suggest that an early functional marker of natriuretic response may provide clinically relevant prognostic information during the initial ED management of ADHF.

This distinction is physiologically important. BNP reflects myocardial wall stress, ventricular filling pressure, and neurohormonal activation, all of which are central to the pathophysiology and risk stratification of heart failure. However, BNP is not a direct marker of the renal response to decongestive therapy, and its concentration may not change sufficiently during the first 2 hours after intravenous diuretic administration to reflect early treatment effectiveness. In contrast, post-diuretic UNa provides a functional readout of loop diuretic activity at the tubular level. Because loop diuretics inhibit sodium reabsorption in the thick ascending limb of the loop of Henle, an early increase in UNa reflects an adequate natriuretic response, whereas a reduced 2-hour UNa concentration may indicate impaired diuretic responsiveness, persistent sodium retention, and ongoing congestion. Therefore, the present findings should not be interpreted as indicating that UNa replaces BNP, but rather that these biomarkers capture different and potentially complementary dimensions of ADHF: cardiac wall stress and early renal natriuretic response.

The prognostic relevance of post-diuretic UNa observed in the present study is consistent with expanding literature emphasizing UNa as an early marker of diuretic efficiency in acute heart failure. In previous studies, lower post-diuretic UNa was associated with insufficient decongestion, longer hospitalization, rehospitalization, and adverse in-hospital outcomes (5,9,10,22,23). However, most available data have been derived from hospitalized cohorts, and many studies have focused on inpatient endpoints or later clinical trajectories rather than the earliest phase of ED management.

The exploratory optimal cutoff value for 2-hour UNa in our cohort was ≤93 mmol/L. This threshold showed moderate sensitivity, relatively high specificity, and acceptable overall accuracy for the composite outcome. From a clinical perspective, such a pattern may be useful for identifying patients who are less likely to achieve an adequate early natriuretic response and may therefore warrant closer observation or more cautious disposition planning. Previous literature has commonly considered post-diuretic UNa values below 70 mmol/L as suggestive of an insufficient natriuretic response and a worse clinical trajectory (16,17,24,25), whereas some authors have proposed higher thresholds, including values around 100 mmol/L (22,26,27), for defining a more satisfactory early diuretic response. The cutoff identified in the present study falls within this broader range, but it should be interpreted cautiously as our study was a relatively small, single-center trial. Therefore, the optimal cutoff value of ≤93 mmol/L should be considered hypothesis-generating rather than a definitive clinical threshold, and it should be externally validated in larger multicenter ED cohorts before it is applied in routine decision-making.

Beyond UNa, our findings also support the relevance of other markers of hemodynamic compromise in short-term adverse outcomes. Patients with the composite outcome had significantly higher baseline lactate levels and were more likely to present with a wet-cold clinical profile. In ADHF, the most common hemodynamic presentation is generally the wet-warm profile, reflecting the predominance of congestion in patients who still maintain relatively preserved peripheral perfusion (28). The wet-cold profile is usually less frequent but clinically more concerning, as it reflects the coexistence of congestion and impaired tissue perfusion, often indicating a lower cardiac output state and more advanced hemodynamic derangement (28). The higher prevalence of wet-cold patients in our event group therefore appears pathophysiologically consistent with the worse short-term outcomes observed in these individuals. In parallel, elevated lactate may reflect reduced effective forward flow and systemic hypoperfusion even when overt shock is not present (29). Taken together, these findings suggest that poor short-term prognosis in ADHF is driven not only by persistent congestion but also by the simultaneous presence of circulatory insufficiency, and that the wet-cold profile and lactate may provide clinically relevant complementary information alongside UNa.

Similarly, higher admission hs-cTnI levels were associated with adverse outcomes in univariable analysis. This is also biologically plausible, as myocardial injury in acute heart failure may reflect subendocardial ischemia, increased wall stress, or concomitant myocardial damage, all of which are known to portend worse prognosis (30). In the present study, hs-cTnI remained independently associated with the composite outcome in the adjusted model, which suggests that myocardial injury may provide prognostic information beyond clinical congestion and natriuretic response. Nevertheless, the association between 2-hour UNa and the outcome remained significant after adjustment for hs-cTnI and other clinically relevant variables, which supports the independent contribution of early natriuretic response to short-term risk stratification.

Another relevant result was the association between higher VExUS grades and the composite outcome. VExUS provides a structured sonographic assessment of systemic venous congestion and, unlike conventional echocardiographic parameters such as ejection fraction, may better capture the hemodynamic burden of venous overload on end organs (31). In our study, ejection fraction did not differ significantly between the groups, whereas VExUS severity did. This distinction is important because short-term outcomes in ADHF are often driven less by left ventricular systolic function alone and more by the overall congestion-perfusion profile (28). Accordingly, our data suggest that functional markers of decongestive response, such as UNa, and anatomical/hemodynamic markers of venous congestion, such as VExUS, may provide complementary information for early risk assessment.

Several study limitations should be acknowledged. First, this was a single-center study with a relatively modest sample size, which may limit the generalizability of the findings. Second, the composite endpoint combined several clinically relevant short-term adverse outcomes, including ICU admission at the index visit, ED revisit, and 30-day mortality. Although this approach allowed a broader assessment of early clinical deterioration, the individual components may reflect partially different clinical trajectories. In particular, ICU admission may be influenced by institutional practice patterns and physician discretion, whereas ED revisit may be affected by discharge strategy, follow-up availability, and health care access. Therefore, the composite outcome should be interpreted as an overall marker of a short-term adverse clinical course rather than as a uniform measure of disease severity. Third, although multivariable logistic regression analysis was performed, the relatively modest sample size and limited number of events may have affected the robustness and stability of the adjusted model. Therefore, the multivariable findings should be interpreted with caution and considered exploratory rather than definitive. Fourth, treatment decisions were made by the attending physicians as part of routine clinical care and were not strictly protocolized by the study, which may have introduced some management-related heterogeneity. Fifth, detailed baseline heart failure therapy, including the full spectrum of guideline-directed medical therapy, was not systematically evaluated. Because background heart failure therapy and previous diuretic exposure may influence the early natriuretic response, this issue should be considered when interpreting the association between 2-hour UNa and short-term outcomes. Finally, follow-up was limited to 30 days, in keeping with the study’s focus on short-term prognosis; therefore, the findings should not be directly extrapolated to longer-term outcomes.

Taken together, our findings may have practical implications for early ED-based risk assessment in ADHF. Rather than suggesting that UNa should replace BNP, our results support a complementary biomarker approach. BNP remains an established marker of cardiac wall stress and overall heart failure burden, whereas 2-hour UNa provides early information on the adequacy of renal natriuretic response after intravenous loop diuretic therapy. In this context, a low 2-hour UNa may help identify patients who require closer reassessment of decongestion, renal response, and disposition planning during the initial phase of ED management. Importantly, early UNa should not be interpreted in isolation, but together with clinical phenotype, perfusion markers such as lactate, myocardial injury markers such as hs-cTnI, hemodynamic profile, and congestion assessment tools such as VExUS. Therefore, the clinical value of 2-hour UNa may lie not in replacing conventional biomarkers but in adding a treatment-response dimension to early risk stratification in ADHF.
